# Dopaminergic Input to the Inferior Colliculus in Mice

**DOI:** 10.3389/fnana.2015.00168

**Published:** 2016-01-21

**Authors:** Alexander A. Nevue, Cameron J. Elde, David J. Perkel, Christine V. Portfors

**Affiliations:** ^1^School of Biological Sciences, Washington State University VancouverVancouver, WA, USA; ^2^Department of Biology, University of WashingtonSeattle, WA, USA; ^3^Department of Otolaryngology—Head and Neck Surgery, University of WashingtonSeattle, WA, USA; ^4^The Virginia Merrill Bloedel Hearing Research Center, University of WashingtonSeattle, WA, USA

**Keywords:** subparafascicular thalamic nucleus, tyrosine hydroxylase, auditory midbrain, catecholamines, tract tracing

## Abstract

The response of sensory neurons to stimuli can be modulated by a variety of factors including attention, emotion, behavioral context, and disorders involving neuromodulatory systems. For example, patients with Parkinson’s disease (PD) have disordered speech processing, suggesting that dopamine alters normal representation of these salient sounds. Understanding the mechanisms by which dopamine modulates auditory processing is thus an important goal. The principal auditory midbrain nucleus, the inferior colliculus (IC), is a likely location for dopaminergic modulation of auditory processing because it contains dopamine receptors and nerve terminals immunoreactive for tyrosine hydroxylase (TH), the rate-limiting enzyme in dopamine synthesis. However, the sources of dopaminergic input to the IC are unknown. In this study, we iontophoretically injected a retrograde tracer into the IC of mice and then stained the tissue for TH. We also immunostained for dopamine beta-hydroxylase (DBH), an enzyme critical for the conversion of dopamine to norepinephrine, to differentiate between dopaminergic and noradrenergic inputs. Retrogradely labeled neurons that were positive for TH were seen bilaterally, with strong ipsilateral dominance, in the subparafascicular thalamic nucleus (SPF). All retrogradely labeled neurons that we observed in other brain regions were TH-negative. Projections from the SPF were confirmed using an anterograde tracer, revealing TH-positive and DBH-negative anterogradely labeled fibers and terminals in the IC. While the functional role of this dopaminergic input to the IC is not yet known, it provides a potential mechanism for context dependent modulation of auditory processing.

## Introduction

Dopamine plays many roles in the brain, in such processes as movement, attention, reward, and motivation, and in disorders including Parkinson’s disease (PD), schizophrenia, and addiction (Maia and Frank, 2011). Less well understood is a role for dopamine in sensory processing, though there is mounting evidence that dopamine is involved in auditory processing. For example, people with PD, a neurological disease caused by degeneration of the dopaminergic system, have difficulties processing speech, including deficits estimating the time intervals of an acoustic speech signal (Gräber et al., 2002), altered emotional prosody (Schröder et al., 2010), and difficulty perceiving the individual’s own loudness (Kwan and Whitehill, 2011). Dopamine modulates auditory responses to vocalizations in songbirds (Leblois et al., 2010) and is present in auditory circuitry in fish (Forlano et al., 2014). In addition, neural responses in the principal auditory midbrain nucleus, the inferior colliculus (IC), can be altered by a reward-based stimulus (Metzger et al., 2006) and by the application of exogenous dopamine (Gittelman et al., 2013). Although it is clear that dopamine modulates auditory processing in the forebrain and midbrain, the source(s) of dopaminergic input to auditory regions are not known.

In this study, we examined the sources of dopaminergic input to the IC. We focused on the IC because it is an obligatory station in the ascending auditory pathway, contains dopaminergic fibers and terminals (Paloff and Usunoff, 2000; Tong et al., 2005), and expresses D2-like dopamine receptors (Wamsley et al., 1989; Weiner et al., 1991; Hurd et al., 2001; Satake et al., 2012). While the majority of dopaminergic neuron somata are located in the substantia nigra and ventral tegmental area, there are many groups of dopaminergic neurons in the central nervous system. One potential candidate for dopaminergic innervation of the IC is the subparafascicular thalamic nucleus (SPF). The SPF is split into two subdivisions, the magnocellular (SPFm) and parvicellular (SPFp; Paxinos and Franklin, 2001) and is part of the A11 dopaminergic cell group (Takada et al., 1988). While the SPF is known to project to the IC (Yasui et al., 1992), it is not known whether these neuronal projections are dopaminergic. Dopaminergic targets of the SPF that have been identified to date include the neocortex, spinal cord, olfactory tubercle, and amygdala (Takada et al., 1988; Takada, 1993) but not the IC (Moriizumi and Hattori, 1992).

The goal of this study was to determine the sources of dopaminergic input to the IC by placing a retrograde tracer into the IC of mice and immunostaining for tyrosine hydroxylase (TH). We found the SPF to be the region where retrogradely labeled neurons were dopaminergic. Anterograde tracer injected in the SPF labeled TH-positive terminals in the IC, indicating that the SPF provides the dopaminergic input to the IC. Determining the source of dopaminergic projections into the auditory system is an important first step in understanding the effects and underlying cellular and synaptic actions of dopamine on auditory processing.

## Materials and Methods

### Animals

We used normal hearing CBA/CaJ and C57BL/6 adult mice (5 males, 8 females) obtained from Jackson Laboratory or bred in our colony for the retrograde (*n* = 9) and anterograde (*n* = 4) tracer experiments. All animals had free access to food and water and were housed on a reversed 12 h light/12 h dark schedule. All care and procedures were in accordance with the guidelines of the National Institutes of Health and were approved by the Washington State University Institutional Animal Care and Use Committee.

### Preparation of Animals for Tracer Injections

We iontophoretically injected the tracers into the regions of interest in awake animals. To prepare the animals for the tracer injections, they were anesthetized for mounting of a headpost onto the skull using surgical techniques we have previously described (Muniak et al., 2012). Once the headpost was mounted, we made a craniotomy (about 1 mm × 1 mm) above the desired brain region based on stereotaxic coordinates (Paxinos and Franklin, 2001). We then covered the hole with petroleum jelly and/or bone wax to prevent the brain from dehydrating, applied lidocaine and an antibiotic (Neosporin) to the exposed muscle, and returned the mouse to its home cage to recover from the surgery for at least 1 day before a tracer deposit was made. On the experimental day, the animal was placed in a sound attenuating chamber with its headpost bolted into a custom stereotaxic apparatus. The animal was given a low dose (<5 mg/kg, i.p.) of acepromazine to ease any stress of putting the animal in the restraint.

### Iontophoretic Tracer Injections

For retrograde tracing, we used a 2–4% solution of Fluorogold (FG; Fluorochrome) in a sodium acetate buffer in nine animals. In two animals, we injected both FG and 1% cholera toxin subunit B (CTB; List Biological Laboratories) dissolved in distilled water into the IC. We used glass micropipettes (resistance 3–5 MΩ) filled with the FG or CTB to record electrophysiological response properties prior to depositing the tracer. Recording electrodes were advanced into the left IC by a hydraulic micropositioner (David Kopf Instruments) driven from outside the sound attenuating chamber. We used standard methods in our laboratory to record extracellular electrical activity in response to auditory stimuli (Gittelman et al., 2013). We deposited the tracers once we were confident that the tip of the electrode was in the IC based on frequency responses of the multiunit clusters (Portfors et al., 2011). The FG and CTB were deposited by injecting 5 μA of current for 8 min (7 s on/7 s off). The animal was then returned to its home cage for 7 days survival time.

In four mice, we deposited 10% 10,000 MW biotinylated dextran amine (BDA; Life Technologies) dissolved in 0.9% saline into the SPF. We located the SPF using stereotaxic coordinates (1.3–1.6 mm caudally from bregma and 0.1–0.5 mm lateral to the midline; Paxinos and Franklin, 2001). The BDA was deposited by injecting 5 μA of current for 10 min (7 s on/7 s off). The animal was then returned to its home cage for 7 days survival time.

### Perfusion and Tissue Collection

The mice were deeply anesthetized with isoflurane in an induction chamber. We then transcardially perfused each mouse using 60 mL of buffered 10% formalin or 4% paraformaldehyde in 0.1 M phosphate buffer solution (PBS, pH 7.4). The brain was removed and cryoprotected overnight in 20% sucrose solution in 0.1 M PBS. We sectioned the brain coronally at a thickness of 50 μm starting just caudal to the dorsal cochlear nucleus and ending rostral to the SPF using a Leica SM2000 R freezing microtome (Leica Biosystems). Sections were collected serially in 0.1 M PBS and stored at 4°C until use.

### Immunohistochemistry; TH, DBH, and Streptavidin

Sections were rinsed 10× in 0.1 M PBS, then gently shaken while incubated overnight in a solution of rabbit anti-TH polyclonal primary antibody (1:1500, Millipore), 3% normal donkey serum (Millipore), and 0.4% Triton X-100 (Sigma-Aldrich) in 0.1 M PBS. The polyclonal primary antibody for dopamine beta-hydroxylase (DBH) made in a rabbit (1:4000, ImmunoStar) was used for differentiating dopaminergic and noradrenergic inputs. Sections were rinsed 10× in 0.1 M PBS. TH and DBH labeled cells and fibers were visualized by incubating the sections in fluorescently tagged secondary antibodies for 2 h. The secondary antibody used for labeling TH and DBH primary antibodies was either an Alexa Fluor 488 or 568 conjugated to donkey anti-rabbit IgG (1:250, Life Technologies). For confocal microscopy, we used an antibody to FG (1:800, Millipore) with an Alexa Fluor 568 secondary (1:250, Life Technologies) and a sheep anti-TH polyclonal antibody (1:1500, Millipore) with an Alexa Fluor 488 secondary (1:250, Life Technologies). To visualize the CTB we used a goat anti-CTB primary antibody (1:10,000, List Biological Laboratories) with an Alexa Fluor 568 conjugated anti-goat secondary antibody (1:250, Life Technologies). For the anterograde experiments, we used Alexa Fluor 488 conjugated streptavidin (1:250, Life Technologies) to visualize the tracer. The sections were mounted on Superfrost Plus microscope slides (Fisher Scientific), dehydrated and cleared, and coverslipped with DPX (Electron Microscopy Sciences). Label was observed using a Leica TCS SP8 confocal microscope (Leica Microsystems). The location of labeled cell bodies and terminals were identified using a Nissl stain on the adjacent series of sections.

### Antibody Characterization

The specificity of the TH antibody was validated by labeled cells in known dopaminergic areas such as the substantia nigra (Hokfelt et al., 1976), as well as the characteristic shape of the SPFm, defined by the TH-positive cells (Yasui et al., 1992). The specificity of the DBH antibody was shown by the presence of DBH-positive neurons in the locus coeruleus (Benarroch, 2009). Additionally, areas of the brain, including the SPF, that are known to contain dopaminergic and not noradrenergic cell bodies showed positive immunofluorescence for the TH antibody but not the DBH antibody (Takada et al., 1988). Antibodies used in these experiments are given in Table 1.

**Table 1:** **List of antibodies used in this study**.

Level	Antigen	Host	Conjugated	Dilution	Source
Primary	TH	Rabbit	–	1:1500	Millipore AB152
Primary	TH	Sheep	–	1:1500	Millipore AB1542
Primary	DBH	Rabbit	–	1:4000	ImmunoStar 22806
Primary	FG	Rabbit	–	1:800	Millipore AB153-I
Primary	CTB	Goat	–	1:10,000	List Biological #703
Secondary	Anti-rabbit	Donkey	Alexa 488	1:250	Life Technologies A-21206
Secondary	Anti-rabbit	Donkey	Alexa 568	1:250	Life Technologies A-10042
Secondary	Anti-sheep	Donkey	Alexa 488	1:250	Life Technologies A-11015
Secondary	Anti-goat	Donkey	Alexa 568	1:250	Life Technologies A-11057

### Data Analysis

For analysis of double labeling, we examined every other 50 μm section in the SPFm. Approximately four sections were counted for each animal. Cells were counted with live images. In each section, we counted the number of retrogradely labeled cells and the number of retrogradely labeled cells that were TH positive on the ipsilateral and contralateral sides. We used Student’s *t*-tests to determine statistical significance between groups. Data are presented as mean ± *SD*. For subjective analysis of labeled fiber density, an observer assigned a score of 0–3 to fiber density while examining the label in the microscope. A score of 0 represented no fibers or terminals and a score of 3 represented extremely dense terminal labeling.

## Results

**Figure 1:**
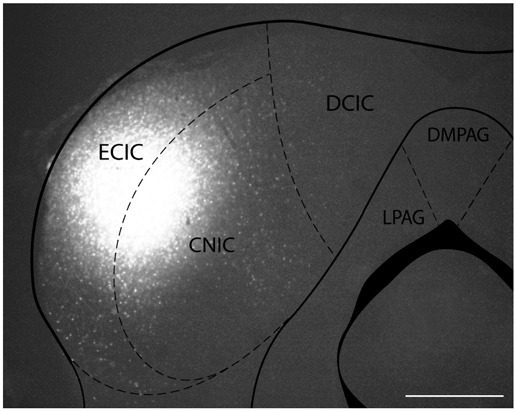
**Iontophoretic injections of Fluorogold (FG) and cholera toxin subunit B (CTB) were made in the left inferior colliculus (IC)**. These injections were guided by electrophysiological response properties in awake animals. All deposits were confirmed to be in the IC. Example deposit site overlaid on a schematic coronal section of the IC (modified from Paxinos and Franklin, 2001) to delineate the boundaries of the IC. Atlas used with permission from Paxinos and Franklin (2001). Abbreviations: CNIC, central nucleus of the IC; ECIC, external cortex of the IC; DCIC, dorsal cortex of the IC. DMPAG and LPAG, periaqueductal gray. Scale bar 250 μm.

To determine the sources of dopaminergic input to the IC, we iontophoretically injected FG or CTB into the left IC in nine mice. In two mice, deposits of both FG and CTB were made. The location of each deposit was confirmed after tissue processing (Figure 1). In all of these animals, the tracer did not spread into neighboring regions such as the superior colliculus or periaqueductal gray. Some of the lateral deposits had a small diffusion into the cerebellum but these deposits were still used for quantification because we did not observe any anterogradely labeled fibers from the SPF in the cerebellum. By staining the same sections for TH and DBH, we identified which of the retrogradely labeled cell bodies were dopaminergic.

**Figure 2:**
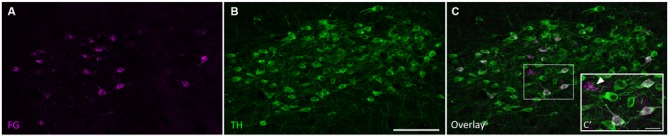
**Tyrosine hydroxylase (TH)-positive cells in the SPF project to the IC. (A)** Retrogradely labeled cells in the SPF after a FG deposit in the IC. **(B)** TH-positive neurons in the SPF. **(C)** Overlay of images showing TH immunoreactivity in retrogradely labeled cells. **(C’)** Inset shows retrogradely labeled neurons stained with TH (magenta+green yields white overlay), and retrogradely labeled TH-negative neurons (marked with an arrow). In this section, 25 cells were retrogradely labeled, 22 of which were TH-positive. Scale bar: **(B)** 100 μm; **(C’)** 20 μm.

Retrogradely labeled cell bodies were found in all the expected auditory nuclei including the dorsal cochlear nucleus, ventral cochlear nucleus, contralateral IC, superior olivary complex, nuclei of the lateral lemniscus, and auditory cortex (Adams, 1979; Brunso-Bechtold et al., 1981; Saldaña et al., 1996; Frisina et al., 1998; Winer et al., 1998). However, none of those labeled cells were TH-positive. Cell bodies containing both FG and TH immunoreactivity, but not DBH immunoreactivity, were found only in the SPF. Few retrogradely labeled and TH-positive cells were found in the rostral part of the SPFp. Strong double labeling was seen in the SPFm (Figure 2).

**Figure 3:**
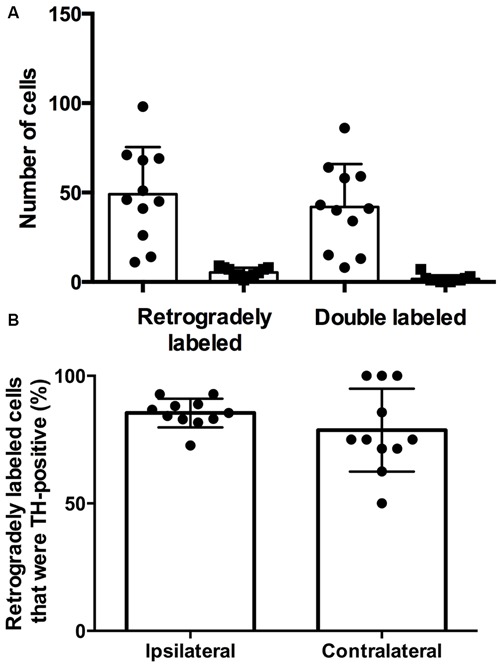
**The majority of retrogradely labeled cells in the SPF were TH-positive. (A)** Number of retrogradely labeled cells in the SPF and number of retrogradely labeled cells that were TH-positive on each side (ipsilateral, circles; contralateral, squares). Each symbol represents data from one deposit in the IC. **(B)** The average percentage of retrogradely labeled cells that were TH-positive in the ipsilateral and contralateral SPF was not significantly different. Each symbol indicates the percentage of retrogradely labeled cells that were TH-positive in the SPF after one deposit in the IC.

There were more retrogradely labeled cells in the ipsilateral SPF than in the contralateral SPF (ipsilateral, 49.1 ± 26.3; contralateral, 5.4 ± 2.6; Figure 3A). Although the ipsilateral side had a significantly higher number of doubly labeled cells than the contralateral side (ipsilateral, 41.9 ± 24.0; contralateral, 1.6 ± 2.0; *p* < 0.001), the percentage of retrogradely labeled cells that were TH-positive was not significantly different between the ipsilateral and the contralateral side (85.4 ± 5.6% vs. 78.73 ± 16.3%; *p* = 0.212; Figure 3B).

**Figure 4:**
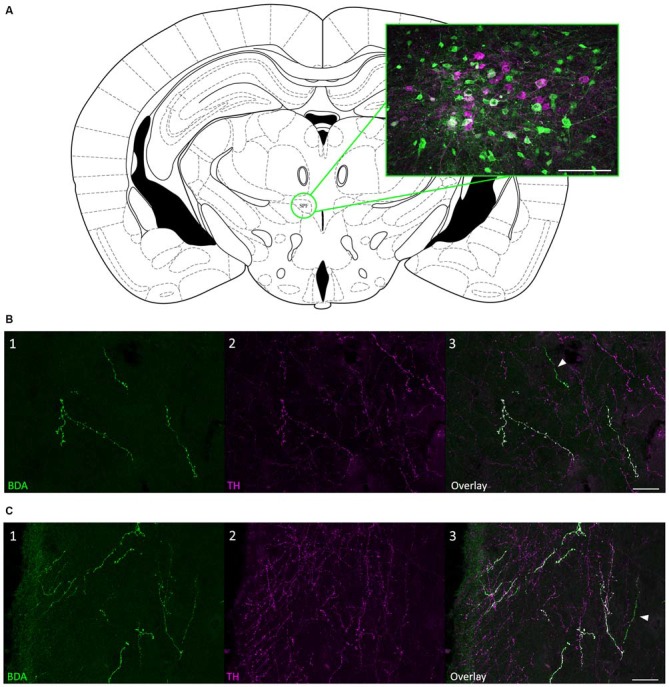
**Anterograde tracing confirmed that projections from SPF to IC were TH-positive. (A)** Coronal section showing the SPF and an example biotinylated dextran amine (BDA) deposit located within the SPF. The magenta cells were TH-positive; green cells were labeled with BDA. **(B)** Anterogradely labeled fibers from the SPF were colocalized with TH in the CNIC. **(B1)** Anterogradely labeled fibers and terminals in the CNIC, ipsilateral to the deposit site. **(B2)** TH-positive fibers and terminals in the ipsilateral CNIC. **(B3)** Overlay of images showing that some anterogradely labeled fibers were TH-positive (white) in the ipsilateral CNIC. The arrow denotes an anterogradely labeled fiber that was TH-negative. **(C)** Anterogradely labeled fibers from the SPF were colocalized with TH in the ECIC. **(C1)** Anterogradely labeled fibers and terminals in the ECIC, ipsilateral to the deposit site. **(C2)** TH-positive fibers and terminals in the ipsilateral ECIC. **(C3)** Overlay of images showing that some anterogradely labeled fibers were TH-positive (white) in the ipsilateral ECIC. The arrow denotes an anterogradely labeled fiber that was TH-negative. Atlas used with permission from Paxinos and Franklin (2001). Scale bar: **(A)** 100 μm; **(B,C)** 25 μm.

**Figure 5:**
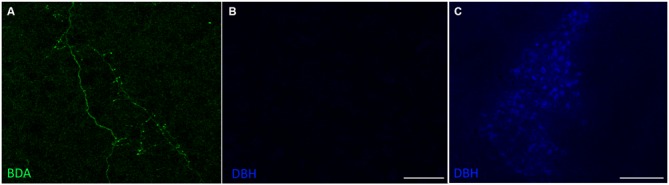
**Projections from the SPF to the IC were dopamine beta-hydroxylase (DBH)-negative. (A)** Anterogradely labeled fibers and terminals in the CNIC, ipsilateral to the deposit site. **(B)** Anterogradely labeled fibers and terminals in the CNIC were DBH-negative. **(C)** DBH-positive cells in the locus coeruleus served as an internal positive control for the DBH antibody. Scale bar: **(B)** 50 μm; **(C)** 100 μm.

To confirm that the SPF sends dopaminergic projections to the IC, we injected 10K BDA, an anterograde tracer, into the SPF in four mice (Figure 4A). We found anterogradely labeled fibers that were TH-positive in both the ipsilateral and contralateral IC. Additionally, there were anterogradely labeled fibers in the IC that were TH-negative, which is in agreement with the results of our retrograde tracing. Anterogradely labeled fibers that were both TH-positive and TH-negative were found in the central nucleus (Figure 4B), and external and dorsal cortices of the IC (Figure 4C). Qualitatively, there were more anterogradely labeled fibers from the SPF that were TH positive in the external and dorsal cortices of the IC than in the central nucleus (Table 2). An important future study will be to determine the topography of the projections from SPF to IC and determine the spatial distribution in subdivisions of the IC. Anterogradely labeled fibers in the IC were DBH-negative (Figure 5). Although DBH immunoreactivity has not been detected in SPF somata, it is possible that their terminals in IC do express DBH and could synthesize norepinephrine. We tested this possibility, and did not find DBH immunoreactivity in SPF-derived terminals within the IC, confirming that the TH-positive fibers were dopaminergic but not noradrenergic. There were TH-positive fibers in the IC that were not labeled with BDA. These could be unlabeled afferent fibers from SPF, or they could represent noradrenergic fibers from the locus coeruleus (Klepper and Herbert, 1991; Hormigo et al., 2012). No TH-positive cell bodies were observed in the IC (Jaeger and Joh, 1983; Kitahama et al., 1996).

**Table 2:** **Qualitative distribution of TH-positive fibers in the inferior colliculus (IC)**.

Subdivision of IC	Density of TH fibers	Density of TH + BDA fibers
CNIC	++	+
ECIC	+++	++
DCIC	+++	++

*Note: + indicates low density and +++ indicates high density*.

**Figure 6:**
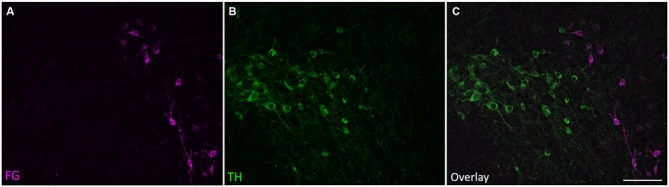
**Retrogradely labeled cells outside the SPF were TH-negative**. Retrogradely labeled cells in the ipsilateral substantia nigra pars lateralis were TH-negative. **(A)** Retrogradely labeled cells in the substantia nigra after an IC deposit. **(B)** TH-positive cells in the substantia nigra. **(C)** Overlay of images showing that the retrogradely labeled cells in the substantia nigra were not TH-positive. Scale bar 100 μm.

Finally, we found that all other retrogradely labeled cells were TH-negative, including those in known dopaminergic areas such as the substantia nigra (Figure 6). As expected, noradrenergic cells in the locus coeruleus were retrogradely labeled (Klepper and Herbert, 1991; Hormigo et al., 2012).

## Discussion

Although the IC contains dopamine receptors (Wamsley et al., 1989; Weiner et al., 1991), has TH-positive fibers (this study; Tong et al., 2005), and shows altered neuronal response properties when exogenous dopamine is applied (Gittelman et al., 2013), the source of the dopaminergic innervation in the IC has not yet been identified. In this study, we combined retrograde tract tracing with immunostaining for TH and found that the SPF is the major source of dopaminergic input to the IC of mice. Knowing the source of the dopaminergic input to the IC is the first step in determining the behavioral contexts that elicit dopamine release in the IC, and will facilitate future studies to tease apart the neuromodulatory circuits involved in the encoding of salient auditory stimuli.

Prior to this study, it was not known that dopaminergic neurons in the SPF project to the auditory system. We found that approximately 80% of the retrogradely labeled cells in the SPF were TH-positive and DBH-negative. Moreover, our anterograde tracer deposits in the SPF resulted in labeled fibers and terminals in the IC that were also TH-positive and DBH-negative, confirming that dopaminergic cells in the SPF project to the IC. The SPF is known to project to the IC, but the cells that project to the IC were previously thought not to be the dopaminergic cell population (Moriizumi and Hattori, 1992). The SPF is known to contain dopaminergic neurons, is part of the A11 dopaminergic cell group (Takada et al., 1988), and sends dopaminergic projections to the spinal cord (Takada, 1993). Animal studies have suggested that these projections are involved in locomotion, pain control, migraines, and restless legs syndrome (Blessing and Chalmers, 1979; Lindvall et al., 1983; Skagerberg and Lindvall, 1985; Clemens et al., 2006; Charbit et al., 2009; Koblinger et al., 2014).

A previous study examined whether dopaminergic neurons in the SPF project to the IC in the rat (Moriizumi and Hattori, 1992). In contrast to our results, they did not find TH immunoreactivity in retrogradely labeled cells in the SPF. It is unclear why that study did not detect TH immunofluorescence in retrogradely labeled SPF neurons projecting to the IC, but one possibility is that the methods used in our study were more sensitive. Our use of confocal imaging and overlaid images ensured that the observed tracer label and TH staining occurred in the same cells. Additionally, the combination of retrograde and anterograde experiments strengthens our results; both clearly showed that dopaminergic neurons in the SPF project to the IC. We are also confident in our tracer deposits because retrogradely labeled cells were found in known auditory structures that project to the IC (Adams, 1979; Brunso-Bechtold et al., 1981; Saldaña et al., 1996; Frisina et al., 1998; Winer et al., 1998), our TH antibody labeled cells in known dopaminergic areas such as the substantia nigra (Hokfelt et al., 1976), and DBH-positive cells were found in the locus coeruleus (Benarroch, 2009).

The SPF is the only nucleus where we found retrogradely labeled, TH-positive, and DBH-negative cells after deposits in the IC. Similar to results of Olazábal and Moore (1989), we found retrogradely labeled cells in the substantia nigra, a known source of dopamine. Although these cells were similar in size and morphology to those that were TH-positive in the substantia nigra and were in close proximity, we did not detect TH immunoreactivity in them. These cells in the substantia nigra are likely GABAergic (Chevalier et al., 1981; Yasui et al., 1991). Thus, the impairments in processing of emotional cues in speech observed in people with PD (Schröder et al., 2010) are not due to a direct dopaminergic connection from the substantia nigra to the IC. It is possible that the loss of dopamine in the substantia nigra affects auditory processing via a more indirect route that may or may not include the IC. The substantia nigra sends dopaminergic projections to the amygdala (Loughlin and Fallon, 1983), and this may modulate emotional speech processing via the projection from the amygdala to the IC (Marsh et al., 2002).

Yasui et al. (1992) proposed that the SPF may be a central relay nucleus for auditory structures. Not only does the SPF project to the IC, it also projects to the auditory brainstem. It receives input from auditory cortex, auditory thalamus, the superior olivary complex, and from the external and dorsal cortices of IC (LeDoux et al., 1985; Yasui et al., 1990, 1992; Wang et al., 2006). The function of these connections is not yet understood. Neurons of the SPF could release other neurotransmitters besides dopamine, including neuropeptides, allowing a richer repertoire of neuromodulatory signaling. In addition to dopamine, the SPF is known to contain enkephalin, somatostatin, substance P, GABA, and prostaglandins (Wamsley et al., 1980; Graybiel and Elde, 1983; Sugimoto et al., 1984; Kosaka et al., 1987a; Breeder et al., 1995). Currently, it is only known that the SPF sends GABAergic projections to the IC (Moriizumi and Hattori, 1992), although these neurons are not the same neurons as the dopaminergic projections (Kosaka et al., 1987b), and that the SPF sends dopaminergic projections to the IC (this study). Based on these projections, the SPF may play a role in modulating neural responses in the IC, and likely other auditory structures. While it is unknown if the SPF responds to sound, the SPF does show *c-fos* activation after audiogenic stress (Palkovits et al., 2004).

The role dopamine plays in directly modulating neural responses to auditory stimuli has been examined in the IC only by applying exogenous dopamine (Gittelman et al., 2013). The IC contains D2-like dopamine receptors (Wamsley et al., 1989; Weiner et al., 1991) and activating these receptors affects the magnitude and timing of neuronal responses to sounds. Based on the results of the current study, we suggest that the dopaminergic projection from the SPF to the IC plays a role in modulating auditory responses.

The behavioral function of this dopaminergic modulation in auditory functioning is unknown. One possible role may be related to auditory attention. For example, in humans, dopamine is associated with the attentional control of auditory perception (Li et al., 2013). Dopamine may also play a role in the encoding of salience of behaviorally relevant sounds. Because dopamine is involved in attributing salience to reward-related stimuli (Berridge and Robinson, 1998) and neurons in the IC of mice have response properties specific to behaviorally relevant stimuli (Holmstrom et al., 2010), dopamine may differentially alter the encoding of salient sounds in the IC. The role the SPF plays in modulating auditory response properties is a rich avenue for future research.

## Author Contributions

All authors had full access to all the data in the study and take responsibility for the integrity of the data and the accuracy of the data analysis. Study concept and design: DJP and CVP. Acquisition of data: AAN and CJE. Analysis and interpretation of data: AAN, DJP, and CVP. Drafting of the manuscript: AAN, DJP, and CVP. Obtained funding: DJP and CVP.

## Conflict of Interest Statement

The authors declare that the research was conducted in the absence of any commercial or financial relationships that could be construed as a potential conflict of interest.
